# Modeling Vehicle Collision Angle in Traffic Crashes Based on Three-Dimensional Laser Scanning Data

**DOI:** 10.3390/s17030482

**Published:** 2017-02-28

**Authors:** Nengchao Lyu, Gang Huang, Chaozhong Wu, Zhicheng Duan, Pingfan Li

**Affiliations:** 1 Intelligent Transportation Systems Research Center, Wuhan University of Technology, Wuhan 430063, China; lnc@whut.edu.cn (N.L.); dzc@whut.edu.cn (Z.D.); 2 Engineering Research Center for Transportation Safety, Ministry of Education, Wuhan 430063, China; 3 Traffic Management Research Institute of the Ministry of Public Security, Wuxi 214151, China; hgtmri@126.com (G.H.); lpfnew@163.com (P.L.)

**Keywords:** traffic safety, traffic accident form, 3D reconstruction, points cloud, collision angle

## Abstract

In road traffic accidents, the analysis of a vehicle’s collision angle plays a key role in identifying a traffic accident’s form and cause. However, because accurate estimation of vehicle collision angle involves many factors, it is difficult to accurately determine it in cases in which less physical evidence is available and there is a lack of monitoring. This paper establishes the mathematical relation model between collision angle, deformation, and normal vector in the collision region according to the equations of particle deformation and force in Hooke’s law of classical mechanics. At the same time, the surface reconstruction method suitable for a normal vector solution is studied. Finally, the estimation model of vehicle collision angle is presented. In order to verify the correctness of the model, verification of multi-angle collision experiments and sensitivity analysis of laser scanning precision for the angle have been carried out using three-dimensional (3D) data obtained by a 3D laser scanner in the collision deformation zone. Under the conditions with which the model has been defined, validation results show that the collision angle is a result of the weighted synthesis of the normal vector of the collision point and the weight value is the deformation of the collision point corresponding to normal vectors. These conclusions prove the applicability of the model. The collision angle model proposed in this paper can be used as the theoretical basis for traffic accident identification and cause analysis. It can also be used as a theoretical reference for the study of the impact deformation of elastic materials.

## 1. Introduction

Research into vehicle collisions has rapidly increased with the more frequent occurrence of traffic accidents [1,2]. In traffic accidents, the vehicle’s collision angle is the important feature to describe the accident’s form, and it is difficult to acquire the vehicle’s collision angle without surveillance video. A vehicular collision is complicated and traditional methods of accident investigation, such as inspection of the vehicle’s body, trace fixing, and photographing the vehicle, do not provide accurate information regarding collision angle. However, accurate estimation of the collision angle is the key for reconstructing an accident; therefore, it is necessary to develop the most accurate method of estimating a vehicle’s collision angle.

Road traffic accident reproduction technology refers to reproducing the form of the accident process accurately, according to the visible traces, in combination with the description of the accident from witnesses following traffic accidents. Many domestic and international scholars have carried out a great deal of in-depth researches on road traffic accident reappearance. Du [3] proposed a road traffic accident reproduction method based on close shot photogrammetry, which to be used for the geometric measurement of road traffic accidents. Using a two-dimensional photogrammetric method together with the path analysis technique to analyze the actual traffic accident is a common accident reappearance method. Baek et al. [4] proposes an accident reappearance method based on the data from tachographs, in which the speed, longitudinal and lateral accelerated speed, steering angle, driving path information, etc. from tachographs in a sextuple double lane test and a serpentine lane test drive are studied and then recreated in PC-Crash™ software (DSD, Linz, Austria) to compare them to the data for the best path in real vehicle experiment and simulation results. Using tachographic data in accident reappearance studies to obtain the optimal driving data leading to a collision is relatively new in auxiliary driving research, but it has significance. In order to solve the problems in simulation experiments of vehicle continuous collisions, Lang et al. [5] developed a vehicle continuous collision accident reappearance system by studying the mechanism of vehicle continuous collision, using the trajectory prediction iterative algorithm, refactoring localization algorithm of contact position, inverse association with the accident, etc., which provides a good reference value for collision angle. Dejan et al. [6] points out that due to the problems of perspective, fuzziness, low brightness, etc. in digital images of traffic accidents, there is significant difficulty in representing traffic accidents based on image data, so the image analysis method has been studied to ascertain if it could be a suitable tool in accident reappearance studies. Michael et al. [7] proposes a method based on a multivariate Monte Carlo solution, setting speed, braking, steering, and many other factors as the variables for input, and using the Monte Carlo method to test the form of the movement before the accident, thus innovatively applying the method to traffic accident reappearance. Becker et al. [8] submitted that reconstruction of the car seat position after a traffic accident has a certain guiding significance.

Related research into methods of accurately predicting collision angle has been undertaken globally. Hermann et al. [9] introduced the parameter settings of vehicle dynamics related to vehicle collision angle in great detail in order to provide references for use in PC-Crash. Since EBS (Equivalent Barrier Speed)-lib requires high-precision inputs of the vehicle’s moving parameters before a collision when it is used for calculating collision angle, imprecise inputs of the speed, steering, braking, etc. will result in unacceptable error. However, the aforementioned parameters are difficult to acquire precisely in traffic accidents. Tabacu et al. [10] built a vehicle collision dynamics model that describes the changes in vehicle body structure before and after a collision, and the study produced accelerated speed and displacement data under different collision angles and car body stiffness values. While this model is not suitable for collision angle calculations, Abdel-Aty et al. [11] studied the critical collision angle of light-duty trucks and established a death time series model of fatal collision angle using traffic accident death statistics. Bogdanovic et al. [12] studied the influence of different input parameters on the simulation results of a vehicle crash and determined that collision angle is one of the most important factors affecting the simulation’s accuracy. Such work confirms the significance of accurate acquisition of vehicle collision angle, which is also the objective of this paper. Car collision angle analysis is an integral part of the study of vehicle collisions, which could make accident reconstruction more accurate than early car crash studies that relied on crash tests alone.

The above-discussed research methods are the ones most commonly adopted by traffic safety researchers in accident reappearance studies, but they are sensitive to initial conditions, and more material evidence about the collision is needed. In the actual identification of an accident, there is no need to trace material evidence beyond vehicle collision deformation. Therefore, based on vehicle collision deformation, the analysis of collision angle can provide support for the traffic accident identification in some cases. Recently, using laser radar to determine vehicle collision deformation has become possible. More in-depth research on 3D reconstruction technology has been conducted across the globe, and the application stage has been reached. In the field of laser 3D scanning reconstruction, Leica (Heerbrugg, Switzerland), 3D DIGITAL and FARO (Sarasota, FL, USA), RIGEL (Horn, Austria), Minolta (Tokyo, Japan), OpTech (Vaughan, ON, Canada), among others, have developed applications that facilitate fast acquisition of large amounts of spatial 3D data. As for 3D reconstruction scanning equipment, the major producers emphasize different indexes, including ranging precision, ranging scope, data sampling rate, the distance between the minimum point, modeling point positioning accuracy, size of the laser spot, scanning field, laser level, and laser wavelength. On the whole, this equipment provides good results, and the original scan data acquired can better express the 3D characteristics of an object. In terms of data processing, El-Hakim et al. [13] established a hardware platform to build 3D modeling systems of indoor scenes. Sequeira et al. [14] built a 3D scene model reconstruction system called AEST (Automatic Environmental Sensor for Telepresence), which integrates many types of 3D reconstruction algorithms including triangulation of three-dimensional point cloud data, data registration, data fusion, etc. It can be used in production management in the construction industry and in modeling the real structures for virtual reality applications. Stamos et al. [15] built a complete laser 3D reconstruction system; the inputs of the system are the untreated sequence of 3D laser scanning scenes and some untreated 2D image sequences under the same scenario, while the output is the scene with real texture mapping for a geometric model. Stamos et al. [16] applied the research results to the 3D reconstruction of historic buildings. All of these studies show that the vehicle collision deformation information required for accident appraisal can be obtained by laser radar.

However, the specifics of gaining deformation information based on the scanning point cloud and then using the deformation characteristics to obtain the vehicle collision angle presents a new problem worthy of study. Vehicle collision angle estimation is currently still in the qualitative analysis stage, generally, as the known input parameters of a collision study. Only a few studies separately discuss the accurate calculation of vehicle collision angle. The purpose of this paper is to compensate for the inadequacy of existing research and establish a mathematical model for the calculation of vehicle collision angle in traffic accidents. The collision angle in traffic accidents is critical to master the form of the accident and determine the responsible party of the traffic accident. Moreover, the proposed model can be regarded as the basic model for calculating a vehicle’s collision angle, and it provides a new reference for ascertaining the cause of a vehicle collision in the absence of surveillance video.

## 2. Issue Description and Modeling

### 2.1. Vehicle Collision and the Relevant Hypothesis

When a vehicle’s outer surface presents elastic deformation after being impacted by an external force in a traffic accident, the directing of the external force is the key point to consider in confirming the collision angle. Hooke’s Law indicates that the force of a particle is the product of the particle’s spring constants and the particle’s displacement when the particle is elastically deformed, and the particle’s force direction is the same as the displacement, expressed as

*F* = −*kx*, (1)

where *F* is the particle’s force, *k* is the spring constant or rigidity, and *x* is the deformation after sustaining stress [17].

In this paper, when studying the vehicle’s collision angle, the region of the collision can be regarded as a finite elastomer. When the vehicle crashes, each particle incurs a certain displacement along the stress direction. Therefore, the analysis of a vehicle’s collision angle can be discretized into the direction of each particle’s stress in the collision region.

### 2.2. Modeling

Formula (1) describes the relationship between the single particle’s sufferance force and deformation, as a collision produces a certain area of deformation. Firstly, the collision region is dispersed into finite areas of similar size. When the vehicle is impacted by the force, it can be interpreted that the force is dispersed in the centroids of the dispersed areas. The dispersed centroids’ stress that is sustained can be expressed as

(2) $$\vec{F}=\sum (-k_{i}\vec{x_{i}})$$

where $k_{i}$ is the *i*th dispersed centroids’ rigidity and $\vec{x_{i}}$ is the *i*th dispersed centroids’ deformation.

The forced direction is considered only, while the value of force is not considered. Thus, the collision angle of the collision region, based on Formula (2), can be expressed as

(3) $$\vec{\lambda_{c}}=\sum_{i=1}^{n}k_{i}\cdot \varepsilon_{i}\cdot \vec{\lambda_{i}}$$

where $\vec{\lambda_{c}}$ is the collision surface’s collision angle, $\vec{\lambda_{i}}$ is the *i*th collision point’s normal vector, and $\varepsilon_{i}$ is the *i*th collision point’s deformation parameters, which can be regarded as weights.

Because of the complexity of the material, each point’s $k_{i}$ is not the same. Since we aim to create a theoretical model of the collision angle calculation, the model can be simplified. Suppose that all the points in the collision region have the same rigidity and the materials’ density is uniform. Then, Formula (3) can be simplified as

(4) $$\vec{\lambda_{c}}=\sum_{i=1}^{n}\mu_{i}\cdot \vec{\lambda_{i}}$$

where $\mu_{i}$ is the *i*th deformation value after normalizing, which is defined as the distance between a point’s position before and after the collision.

Formula (4) involves two important parameters: the points’ deformation values and normal vectors. In this paper, we use the surface reconstruction approaches to acquire the two parameters, and describe their acquisition in the following sections.

## 3. Reconstruction of the Crashed Surface

In this study, a 3D laser scanner is used to scan the distorted region. The scanned points can disperse the distorted surface into limited points, and then the surface reconstruction approaches can be used to reconstruct triangular tiles based on the dispersed points. The 3D reconstruction approaches are used to calculate the triangle tiles’ deformations and normal vectors. If the surface equation of the collision surface before collision is known, the deformation can be described as the distances between the scanned points and the surface equation. If the surface equation is not known, then a plane equation should be fitted based on the neighborhood points of the collision region; the deformation can be calculated as well.

### 3.1. Delaunay Triangulation and Improvement Based on the Mapping Method

In this paper, the calculation of the normal vectors is based on the surface reconstruction and the scanned points are disposed based on the Delaunay algorithm. However, the 3D Delaunay triangulation in 3D space to form triangular meshes is inefficient and is not applicable for calculating normal vectors. To overcome this limitation, we propose a novel variation on the Delaunay algorithm based on a mapping approach that is applicable for the reconstruction of a vehicle’s collision surface [18].

**Figure 1:**
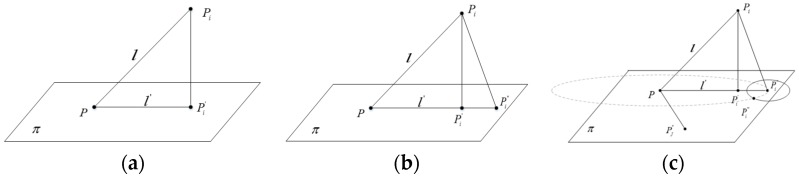
Projection approach with distance adjustment. (**a**) Direct projection; (**b**) First distance adjustment; (**c**) Second distance adjustment.

The basic concept underlying the mapping approach is mapping all the 3D points onto a 2D plane. Considering the superiority of 2D Delaunay triangulation, the mapped points are triangulated in the 2D plane, and then the triangulated result is mapped back to the 3D space to generate the triangulation meshes. The mapping of the points results in the distances’ distortion between the points, as shown in Figure 1a. When mapping point *P_i_*, with the distance l to the point *P*, to the plane π, the point *P_i_* is changed to point $P_{i}^{\prime }$ on the plane π, and the distance between *P* and $P_{i}^{\prime }$ is changed to $l^{\prime }$. Generally, *l* is not equal to $l^{\prime }$ , and so the position of $P_{i}^{\prime }$ should be adjusted after mapping.

The adjustment of the distance is done as follows. $\bar{PP_{i}^{\prime }}$ is extended along the direction of $\bar{PP_{i}^{\prime }}$ to generate a new point $P_{i}^{\prime \prime }$, and $\bar{PP_{i}^{\prime \prime }}$ = $\bar{PP_{i}^{\prime }}$, as shown in Figure 1b. However, this adjustment only ensures that the distance between these two points does not change, and it doesn’t maintain the distance between point *P* and the others. Then, the second distance adjustment is executed. Based on the first adjustment, a circle *O_P_* with radius l centered at point *P* is drawn. The next adjustment position of *P_i_* is on *O_P_*. In addition, the next adjustment position of *P_i_* should meet the distance of another mapping point *P_j_*, as shown in Figure 1c.

Assume that the position of point *P_j_* after the first mapping is located at $P_{j}^{\prime \prime }$. To meet the original distance of $\bar{P_{i}P_{j}}$, another circle is drawn with radius $\bar{P_{i}P_{j}}$ centered at point $P_{i}^{\prime \prime }$. The circle will intersect circle *O_P_* at point $P_{i}^{\prime \prime \prime }$, which is the second adjusted point of *P_i_*. After the mapping process, the mapped point of *P_i_*, namely $P_{i}^{\prime \prime \prime }$, meets the distance of the seed and another point. Then, the mapped point of *P_i_* is adjusted *k* times (*k*th-nearest neighbors, chosen as 5 in this paper) in the same way. After these mappings are done, the mapping position of *P_i_* can be determined by the following formula based on the least-squares method:

(5) $$P_{imap}(P_{it})=\arg\;\min_{\left(P_{it}\right)}\overset{k}{\underset{n=1}{\Sigma }}{\left(d_{P_{it}P_{n}}-d_{P_{i}P_{n}}\right)}^{2}$$

where $P_{it}$ is the calculated mapping position of *P_i_*, $d_{P_{it}P_{n}}$ is the distance between $P_{it}$ and $P_{n}$ in 3D space, and $d_{P_{it}P_{n}}$ is the distance between *P_i_* and *P_n_*.

### 3.2. Analysis of Delaunay Triangulation Based on Improved Mapping Method

The object of this paper is to study the normal vector of the vehicle collision, and the purpose of using the Delaunay triangulation algorithm is to construct a suitable triangular mesh to solve the normal vector of the surface. Therefore, the triangulation mesh is required to be uniform. In order to quantify the result of triangulation in this paper, the variance of the area of all triangles in a triangular mesh is used as the evaluation criterion. In a calculation, such as Formula (6) below, the smaller the variance, the better the result can show that the triangular mesh generation is more suitable for the study described this paper:

(6) $$S^{2}=\frac{1}{n}\sum_{i=1}^{n}(A_{i}-\bar{A}{)}^{2}$$

where $S^{2}$ denotes the triangle area variance, $A_{i}$ the *i*th area of the triangle, $\bar{A}$ the average of all of the areas of the triangle, and *n* the number of triangles.

### 3.3. Impact of Noise on the Triangulation Result

The measurement accuracy of the 3D laser scanner used in this study is ±2 mm. In order to verify the influence of the measurement error of the 3D laser scanner on the triangulation result, we simulated the noise point to verify the applicability of the improved algorithm.

**Figure 2:**
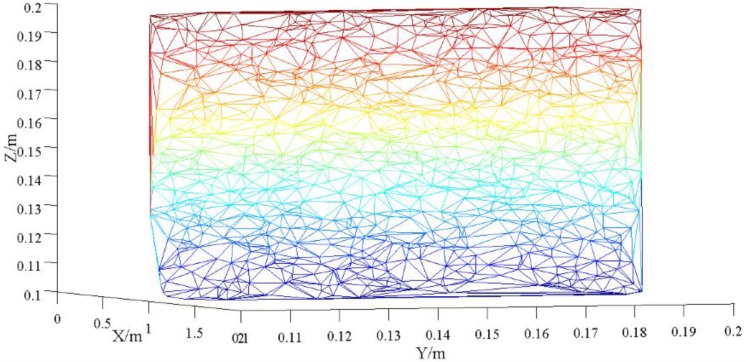
Triangulation of the original point.

According to the scanning characteristics of the 3D laser scanner, we randomly generated 1000 points in the {X=1|Y∈(0.1,0.1),Z∈(0.1,0.2)} plane. As the original data points without noise, the triangulation result is shown in Figure 2.

In Figure 2, there are 1979 triangles in the triangular mesh formed by triangulation. The variance of the area of all triangles is 8.486 × 10^−11^. From the numerical point of view, the triangles formed by triangulation are quite uniform and the space structure is also very good.

In order to study the influence of the noise points within ±2 mm on the triangulation algorithm proposed in this paper, each point is added to the noise of ±0.5, ±1, ±1.5, and ±2 mm. The point used in the theoretical part is to simulate a point scanned by a three-dimensional laser scanner with noise within ±2 mm. The original ideal points are obtained according to the laser scanning rule in an ideal plane, and then random errors were added to these original ideal points [19,20]. Based on these ideal points, random vector in the center of the sphere and random errors in the radius of 0~2 mm were added to generate random errors.

For the Delaunay triangulation based on mapping method for all points, we select the YOZ plane as the projection plane. The variance of the triangle area formed by the triangulation is 7.271 × 10^−11^. It shows that the size of the triangle formed by the triangulation is almost the same, and this result shows that the triangulation result meets the requirements of this paper when the measurement noise is ±0.5 mm.

According to continuous study the results of the triangulation of the measurement error within ±2 mm, we note that the area variance of the triangle in the triangular mesh formed by the triangulation is used as the index to evaluate the quality of the triangulation. The variance of the triangular mesh formed by triangulation with measurement errors of 0.5, 1, 1.5, and 2 mm is shown in Table 1.

From Table 1, we can see that the measurement error within ±2 mm has no significant effect on the mesh size generated by the triangulation. The triangular area variance of the triangular mesh generated by the triangulation is on the order of 10^−11^, and there is no obvious regularity. Therefore, in the triangulation the size of the triangle in the triangular mesh is not sensitive to the measurement error within ±2 mm. In using the mapping method proposed in this paper, in the Delaunay triangulation, the influence of the measurement error within ±2 mm cannot be underestimated.

### 3.4. Analysis of Influence of Projection Surface Selection on the Triangulation Result

The Delaunay triangulation algorithm based on the mapping method has a high requirement for the selection of the projection surface. Inappropriate projection surfaces have a significant impact on the triangulation results, and a split in the triangulation results will affect the calculation of the normal vector to a large extent. Therefore, it is necessary to verify the method of projection in this paper. Therefore, we choose the plane perpendicular to the optical axis of the 3D laser scanner as the projection plane. The optical axis of the 3D laser scanner is the X axis, and the plane perpendicular to it, the YOZ plane, is the best projection plane.

**Figure 3:**
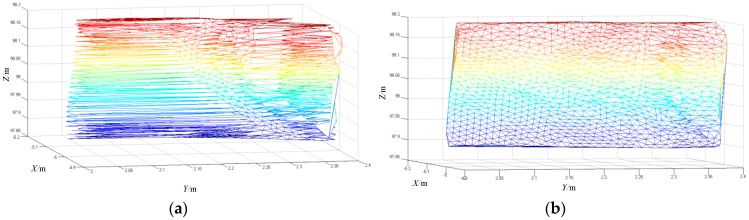
Triangulation results for 3D plane as the projection plane. (**a**) XOZ; (**b**) YOZ.

In Figure 3, there are 2108 and 2112 triangles in the triangle mesh, respectively. There is no significant difference in the number of triangles generated, but there is a significant difference in the quality of the triangular mesh. In Figure 3a, the triangular mesh generated by the left half of the triangle is long and narrow, which seriously does not conform to the rules of triangular formation. This is a result of the failure of the triangulation.

Similarly, the variance of the area of the triangle mesh generated by the triangulation results is 3.153 × 10^−5^. Orders of magnitude have been increased from 10^−5^ to 10^−7^ in Figure 3b, which shows that the triangulation result is a sensitive choice of the projection surface. In order to study the sensitivity of different projections relative to triangulation, in this paper the projection plane X = 0 is rotated along the Z axis to the Y = 0 plane. We also select a projection surface at 15° per interval and calculate the variance of the triangular mesh generated on these projected surfaces. The variance results are shown in Table 2.

It can be seen from Table 2 that in the process of selecting a projection surface from the optimal projection plane to the XOZ plane, which is perpendicular to the optimal projection plane, the uneven degree of the triangular mesh formed by the triangulation is increased exponentially. Therefore, selecting the best projection surface is the key to generating the optimal triangular mesh. In this paper, we determine the optimal projection plane to be the YOZ plane (described as the X = 0 plane). The optical axis of the 3D laser scanner is perpendicular to the YOZ plane, and when using the 3D laser scanner to scan the deformation surface of the vehicle, the display principle of the 3D laser scanner is usually 90° (or approximately 90°) to the optical axis and the impact surface to ensure that there is no overlap between the surfaces of the collision area. Therefore, in this paper, the projection plane is the YOZ plane, which is perpendicular to the optical axis. This ensures that all points obtained by scanning can be projected onto the plane without repetition, and then the triangulation based on the mapping method can proceed.

Summarizing, the quality of the triangular meshes is influenced by point cloud noise, projection plane selection, and projection distance adjustment. The method of projection surface selection, the projection-distance-adjusting method, and the processing of the point cloud noise reduction described in the previous paper that we have proposed here all ensure that the result is the best one when the triangulation is carried out, as it ensures the accuracy of the solution of the normal vector in the following.

## 4. Model Validation and Results Analysis

After completing the triangulation of the point clouds, sparse point clouds were connected into an interrelated triangular mesh. In the case of the known vertex coordinates of each triangle, the normal vector of each triangle can be solved quickly.

### 4.1. Experimental Verification

In order to verify the correctness of the mathematical model of the collision angle calculation, Formula (4), a number of simple collision experiments were designed. The experimental material is aluminum plate of different thicknesses. The uniform density and rigidity of each aluminum plate is constant. A solid iron ball was used as the primary impacting object, and it impacted the aluminum plate at angle of 90°, 75°, 60°, 45°, and 30° to form deformation surfaces.

Taking the 90° frontal impact test as an example, we used a 3D laser scanner in the same position to scan the surface of the aluminum plate before and after impact. After impact deformation, we scanned the aluminum plate following discretization.

We then calculated the deformation of all points in the selected area by MATLAB software. The negative value of the deformation indicates the point “invagination” to the plane.

**Figure 4:**
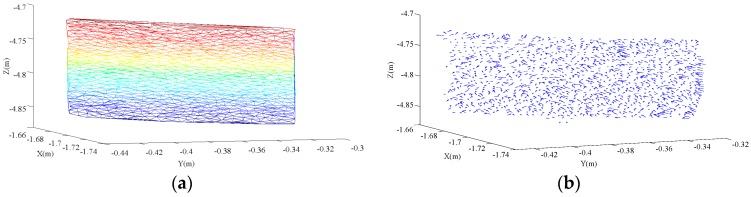
Triangulation and collision points’ normal vectors results. (**a**) Triangulation results; (**b**) Calculation of the collision points’ normal vectors.

The above calculation process obtains the distance from the scanning point after impact to the plane of the aluminum plate before impact. We then proceed to calculate the normal vector of the collision point using the triangulation algorithm proposed in this paper. The triangulation results are shown in Figure 4a, and the normal-vector solution is shown in Figure 4b.

Upon further manual extraction of the normal vectors of the collision points in the collision region, and substituting the corresponding deformation into Formula (4), the normal vector is calculated:

(7) $$\vec{\lambda_{c}}=(0.8195,0.5670,0.0594)$$

Before the collision, the plane of the aluminum plate is

(8) $$\vec{n}=(0.8461,0.5301,0.0551)$$

From Formulas (7) and (8), the angle between the above two quantities can be obtained:

(9) $$<\vec{n},\vec{\lambda_{c}}>=4.3{}^{\circ}$$

The calculated collision angle is 85.7°, which can be considered as the deformation caused by frontal impact. Formula (4) can therefore be used to solve the collision angle in the case of a frontal collision.

The calculation process for other collision angles is the same as the above calculation. The results of several simple impact tests are shown in Table 3.

Analyzing Table 3, we find that the results using the mathematical model, Formula (4), match the collision angle to a high degree of accuracy. When the collision angle is close to 90°, the calculated result is close to the true value. For an aluminum plate with uniform density and rigidity impacted by a solid iron ball at different angles, the collision angle can be calculated based on the simplified model proposed in this paper.

### 4.2. Verification of Real Vehicle Collision Accident

In order to verify the applicability of the model, a real accident is chosen as the object of application analysis.

**Figure 5:**
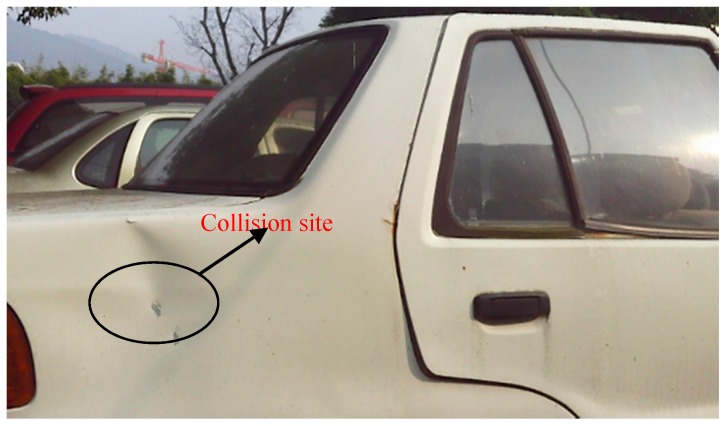
Deformation caused by a real accident.

In the accident, the body of an electric bicycle’s transport cylindrical pile exerts a nearly head-on crush on the rear right-hand side of a private car; the deformation surface of the private car is pictured in Figure 5. Considering that the accident collision deformation surface is small, and that the structural materials of the rear on the left-hand side of the car uses the same material with the same strengh, the collision surface has the characteristics of consistent stiffness and density uniformity. Therefore, all of the above real accident characteristics are in accord with the mathematical model validation.

The method of solving for the normal vector of the collision point is the same as that described in experiment, and the normal vector of the collision area is

(10) $$\vec{\lambda_{c}}=(0.9545,-0.2832,0.0934)$$

Because the impact area is small, the collision plane is approximately considered to be flat. Referring to the edge points of the collision area to match this plane, the normal vector of the matching plane is

(11) $$\vec{n_{rc}}=(0.9694,-0.1823,-0.1646)$$

And the included angle is

(12) $$<\vec{n_{rc}},\vec{\lambda_{c}}>=15.9{}^{\circ}$$

**Figure 6:**
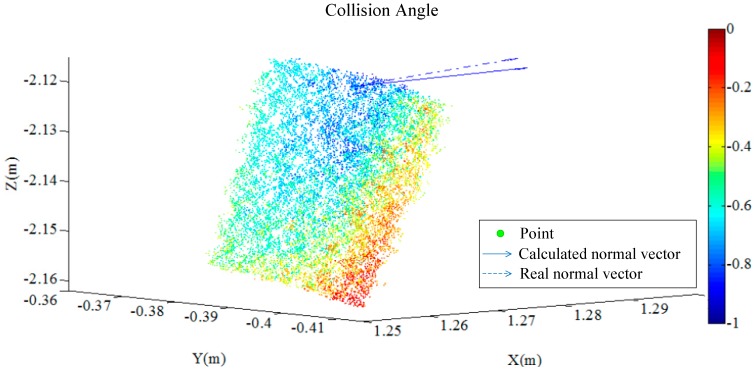
Comparison of the calculated and actual normal vectors.

The calculated results show that the force angle of the rear right-hand side formed by the collision is 74°, and in order to verify the accuracy of the calculation results, according to the comparison of the description from the parties at the scene of the accident and the physical traces of the accident. The cylindrical pile position is restored according to the bundle form at the time of the accident. When the direction of the pile and the bicycle is in the same place, the angle difference is about 11°. When the bicycle movement direction and small car movement direction is vertical, the collision angle between the cylindrical piles and small car is 79°. These conclusions prove that the calculated results are effective, and the collision angle can be calculated. To describe the difference between the normal vector solved in this paper and the collision angle described by the reduction test, we compare the calculation and description methods of the normal vector in the same figure, as shown in Figure 6. The figure shows that the pointing has a good curvature tolerance, and thus the calculated result provides a very good reference value for the analysis and appraisal of a traffic accident.

The collision experiments and the real vehicle collision process have verified the applicability of the collision angle calculation as exemplified by Formula (4) under the conditions that the impact surface has the same stiffness, uniform density, and frontal impact angle, and, compared to other collision calculations, is more accurate. The verified difference between the calculated and actual angles is within 6°, and the experiment is able to facilitate quantitative analysis of the collision direction. Analysis of the real vehicle collision proves that the model is also applicable for collision angle analysis and calculations in traffic accident reconstructions.

## 5. Conclusions

The paper proposes the theoretical model of vehicle collision angle calculation in the accident by using a 3D reconstruction method to solve the key parameters of the model and has the model validation. When the 3D reconstruction technology is applied, the triangulation algorithm based on the mapping method is proposed, and the experimental results show the applicability of the model, which can be applied to the theoretical analysis of the traffic accident vehicle collision angle.

The research methods and conclusions not only provide a new train of thought for the accurate quantitative calculation of the vehicle collision angle in road traffic accidents, they also expand the method of laser point cloud triangle subdivision. However, the proposed model has some limitations: it currently only applies to the calculation of the collision angle in a single object, and a relatively simple deformation surface is required. In order to improve the generality of the model, the next step of research will be carried out in the following aspects:

1. Study the angle of impact from different body material stiffnesses, and have an accurately assignment about the collision point;
2. Propose the correct factors aiming at different deformation surfaces, and make the model suitable for different collision angles’ solving;
3. When it comes to an elongated, groove shape of a collision surface, the collision area can be partitioned for calculation and the research of the collision angle.

## Figures and Tables

**Table 1:** Triangle area variance of triangular mesh generated by different measurement errors.

Error size	0 (no error)	0.5 mm	1 mm	1.5 mm	2 mm
Variance	8.486 × 10^−11^	7.271 × 10^−11^	7.483 × 10^−11^	8.650 × 10^−11^	9.872 × 10^−11^

**Table 2:** Triangular area variance of triangular mesh generated by different projection surfaces.

Plane	YOZ Plane	Rotated 15°	Rotated 30°	Rotated 45°	Rotated 60°	Rotated 75°	XOZ Plane
Variance	1.529 × 10^−7^	3.834 × 10^−7^	9.115 × 10^−7^	2.250 × 10^−6^	5.225 × 10^−6^	1.187 × 10^−6^	3.153 × 10^−5^

**Table 3:** Model verification results of experiments.

Angle (°)	Plane Normal $\vec{n}$	Normal after Collision $\vec{\lambda_{c}}$	Collision Angle (°)	Error (°)
90	(0.8461, 0.5301, 0.0551)	(0.8195, 0.5670, 0.0594)	85.7	4.3
75		(0.7981, 0.4862, 0.3753)	71.1	3.9
60		(0.6985, 0.5389, 0.4709)	64.5	4.5
45		(0.5663, 0.2106, 0.7968)	39.4	5.6
30		(0.3975, 0.2252, −0.8895)	24	6.0

## References

[1] Lu J., Zhang W.J., Yang H.F., Jiang J. (2014). Analysis of Rear-end Risk Based on the Indicator of Time to Collision. J. Transp. Inf. Saf..

[2] Xu S.Y., Lu H.Y., Cheng J. (2015). Design and Implementation of the Vehicle Rear-End Collision Avoidance Terminal System Based on IEEE 802. 11p/1609. J. Transp. Inf. Saf..

[3] Du X., Jin X., Zhang X., Shen J., Hou X. (2009). Geometry features measurement of traffic accident for reconstruction based on close-range photogrammetry. Adv. Eng. Softw..

[4] Se-Ryong B., Joeng-Kwon C., Jong-Jin P., Jong-Han L. (2014). A Reliable Study on the Accident Reconstruction using Accident Data Recorder. J. Inst. Internet Broadcast. Commun..

[5] Lang W., Biao G., Tao C. (2013). Vehicle Continuous Collision Accident Reconstruction System Development. Procedia.

[6] Paliska D., Batista M., Roman S., Dasa F. (2011). An attempt to attain new information in reconstruction of road traffic accidents applying digital image processing. Promet Traffic Transp..

[7] Knox M.A. (2011). Multivariable monte carlo analysis methods in traffic accident reconstruction using python. Proc. ASME Int. Mech. Eng. Congr. Expos..

[8] Becker K., Friedrich K., Rothschild M. (2011). Reconstruction of road traffic accidents. Seating position of automobile occupants. Rechtsmedizin.

[9] Steffan H., Moser A. (1996). The collision and trajectory models of PC-CRASH. SAE Tech. Pap..

[10] Tabacu S., Pandrea N. (2004). Numerical (analytical-based) model for the study of vehicle frontal collision. Int. J. Crashworth..

[11] Abdel M., Abdelwahab H. (2004). Analysis and prediction of traffic fatalities resulting from angle collisions including the effect of vehicles’ configuration and compatibility. Accid. Anal. Prev..

[12] Bogdanovic V., Milutinovic N., Kostic S., Ruskic N. (2004). Research of the influences of input parameters on the result of vehicles collision simulation. Promet Traffic Transp..

[13] Hakim S., Brenner C., Roth C. (1998). A mufti-sensor approach to creating accurate virtual environments. JSPRS J. Photogram. Remote Sens..

[14] Sequeira V., Ng K., Wolfart E. (1999). Automated reconstruction of 3D models from real environments. J. Photogram. Remote Sens..

[15] Stamos I., Allen P.K. (2002). Geometry and Texture Recovery of Scenes of Large Scale. Comput. Vis. Image Underst..

[16] Allen P., Stamos I. 3D modeling of historic sites using range and image data. Proceedings of the IEEE International Conference on Robotics and Automation.

[17] Hu H., Liu F. (2014). Density-functional-theory formulation of classical and quantum Hooke’s law. Sci. China Technol. Sci..

[18] Li F.X., Liu Y.M., Wang X.Z., Rao Y.H. (2015). New Delaunay triangulation algorithm of point cloud based on parametric method. Appl. Res. Comput..

[19] Triglav C.M., Crosilla F.A. (2009). Simplified analytical model for a-priori LiDAR point-positioning error estimation and review of LiDAR error sources. Photogram. Eng. Remote Sens..

[20] Jiang L.F., Lan T., Gu M.X., Ni G.Q. (2012). Effects of laser beamdivergence angle on airborne LiDAR positioning errors. J. Beijing Inst. Technol..

